# Heterologous Biosynthesis of Myxobacterial Antibiotic Miuraenamide A

**DOI:** 10.3390/molecules28062815

**Published:** 2023-03-20

**Authors:** Ying Liu, Satoshi Yamazaki, Makoto Ojika

**Affiliations:** Department of Applied Biosciences, Graduate School of Bioagricultural Sciences, Nagoya University, Nagoya 464-8601, Japan

**Keywords:** miuraenamide A, biosynthesis, heterologous expression, halophilic myxobacteria, *Paraliomyxa miuraensis*

## Abstract

The hard-to-culture slightly halophilic myxobacterium “*Paraliomyxa miuraensis*” SMH-27-4 produces antifungal cyclodepsipeptide miuraenamide A (**1**). Herein, the region (85.9 kbp) containing the biosynthetic gene cluster (BGC) coding the assembly of **1** was identified and heterologously expressed in *Myxococcus xanthus.* A biosynthetic pathway proposed using in silico analysis was verified through the gene disruption of the heterologous transformant. In addition to the core polyketide synthase (PKS) and nonribosomal peptide synthase (NRPS) genes, tyrosine halogenase and *O*-methyltransferase genes participated in the biosynthesis of **1** as their gene-disrupted mutants produced a new congener, debromomiuraenamide A (**4**), and a previously isolated congener, miuraenamide E (**3**), respectively. Multigene disruption provided a heterologous mutant that produced **1** with the highest yield among the prepared mutants. When fed on 3-bromo-L-tyrosine, this mutant produced more **1** in the yield of 1.21 mg/L, which was 20 times higher than that produced by the initially prepared heterologous transformant. Although this yield was comparable to that of the original producer SMH-27-4 (1 mg/L), the culture time was 4.5 times shorter than that of SMH-27-4, indicating a five-fold efficiency in productivity. The results indicate the great potential of the miuraenamide BGC for the future contribution to drug development through logical gene manipulation.

## 1. Introduction

**Figure 1:**
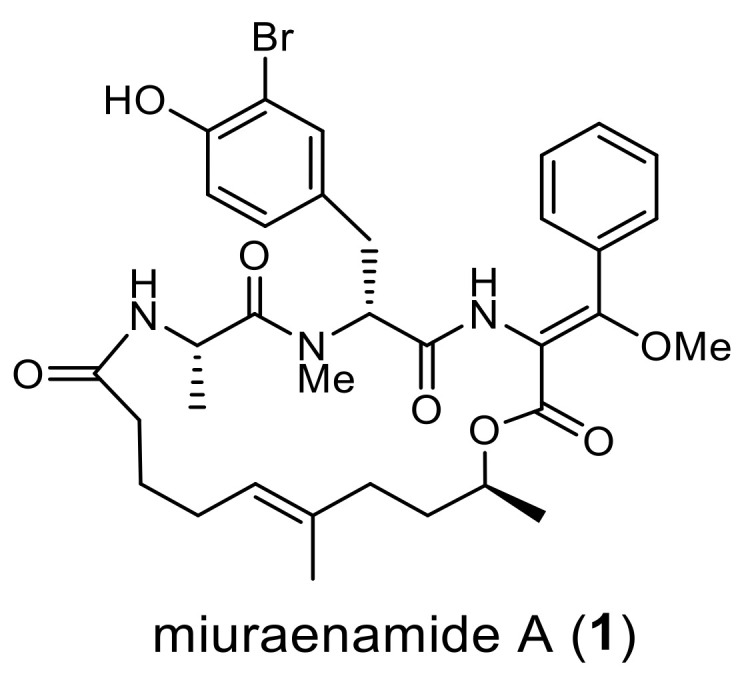
Chemical structure of miuraenamide A (**1**).

Myxobacteria are gram-negative bacteria characterized by gliding, multicellular fruiting body formation and large genome size [1,2,3]. They are considered as good candidates for a next-generation microbial drug factory owing to their potential for producing structurally novel secondary metabolites [4,5,6]. Despite difficulties in isolation and cultivation, a limited number of halophilic strains have been reported and show great potential for producing novel bioactive leads [7,8,9,10]. In 2006, “*Paraliomyxa miuraensis*” SMH-27-4 was isolated from near-seashore soil in Japan [11]. The strain required low salt concentrations of 0.5–1.0% for optimum growth and was regarded as a slightly halophilic myxobacterium. The major secondary metabolite miuraenamide A (**1**, Figure 1) exhibited potent antifungal activity, particularly against the phytopathogenic oomycete *Phytophthora capsici* at a minimum inhibition dose of 25 ng/disk by inhibiting the mitochondrial respiratory chain. Furthermore, it stabilizes actin filaments to change the tumor cell morphology [12]. Cellular studies of **1** reported its effects on cell migration and transcriptional activity [13,14]. It is reported that **1** exhibits a unique actin-binding mode that is different from jasplakinolide, a commonly used pharmacological tool to study actin organization and dynamics in living cells [15]. More recently, **1** has reportedly induced actin filament elongation and shifted the nucleus toward the cell center [16]. Miuraenamide A (**1**) is considered as a new tool that can improve the understanding of the role of actin in living cells. The total synthesis of **1** and the structure-activity studies of several derivatives have also been reported [17,18,19]. We recently analyzed the genome of the strain SMH-27-4 and revealed the presence of 17 biosynthetic machineries for secondary metabolites [20]; however, the detailed biosynthetic mechanism of **1** remains unclear.

The original producer of **1**, SMH-27-4, requires a culture period of 18 days to reach the maximal production (1 mg/L) [11], and is difficult to handle. On seawater agar media, the strain burrows into the agar and does not form distinct single-cell colonies. In liquid broths, the cells aggregate to form cell clusters, rendering obtaining a homogeneous cell suspension impossible. Therefore, the genetic manipulation of the original producer to elucidate the biosynthetic mechanism of **1** is challenging. Herein, we describe the identification and heterologous expression of the biosynthetic gene cluster (BGC) for **1**. The proposed biosynthetic pathway was verified through gene disruption and a multigene region that significantly affected the production of **1** was demonstrated.

## 2. Results

### 2.1. Identification and Heterologous Expression of BGC for Miuraenamide A

**Figure 2:**
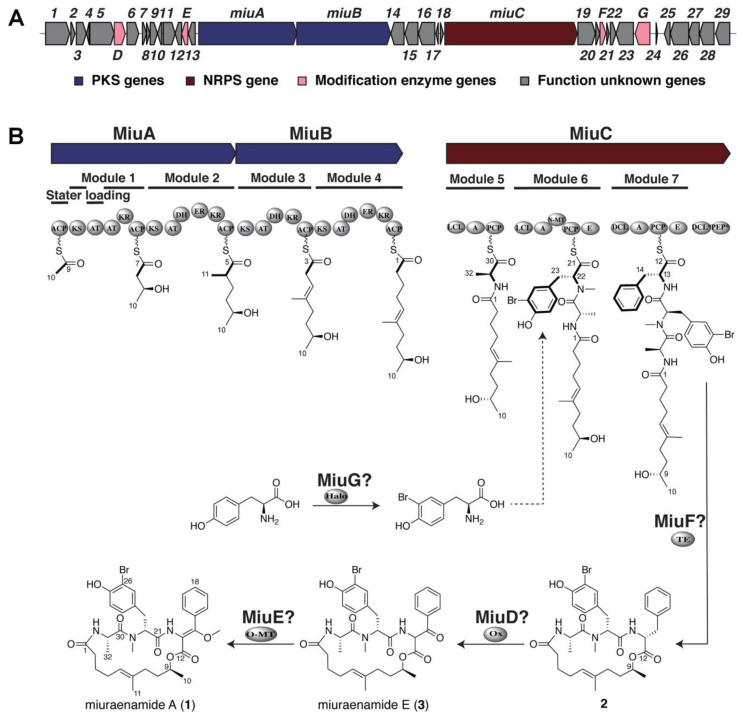
Organization of the *miu* cluster and proposed biosynthetic pathway for miuraenamide A (**1**). (**A**) Map and gene organization of the *miu* cluster-containing region predicted using antiSMASH. (**B**) Proposed biosynthetic pathway of **1**. Abbreviations: ACP, acyl carrier protein; AT, acyl transferase; DH, β-hydroxy dehydratase; ER, enoyl reductase; KR, β-ketoacyl reductase; KS, ketosynthase; A, adenylation domain; PCP, peptidyl carrier protein; E, epimerization domain; O-MT, *O*-methyltransferase domain; N-MT, *N*-methyltransferase domain; LCL, condensation domain that catalyzes the formation of a peptide bond between two L-amino acids; DCL, condensation domain that links an L-amino acid to a growing peptide ending with a D-amino acid; PEP, phosphoenolpyruvate synthase domain; TE, thioesterase; Ox, oxygenase; Halo, halogenase. DCL* and PEP*, DCL and PEP domains unassigned in the proposed biosynthetic mechanism.

The backbone of **1** can be divided into a five-unit polyketide and a tripeptide composing alanine, bromotyrosine, and methoxylated phenylalanine, suggesting that it is a hybrid metabolite of the polyketide synthase-nonribosomal peptide synthase (PKS-NRPS) type. The draft genome of “*P. miuraensis*” SMH-27-4 [20] was analyzed using antiSMASH to find a PKS-NRPS type gene cluster that possessed the predicted substrate selectivity of the constituent modules and the assembly order matching the backbone of **1**. Therefore, this cluster was considered to contain the whole BGC for **1** (*miu* cluster), which spread over the tentative range of 85.9 kbp containing 36 open reading frames (orfs) (Figure 2A). It is rare that there are several function-unknown orfs (e.g., *orf14–18*) that break the continuity of the core (PKS and NRPS) genes. We found only two BGCs of this type among 82 PKS-containing myxobacterial BGCs in the MiBIG database.

**Figure 3:**
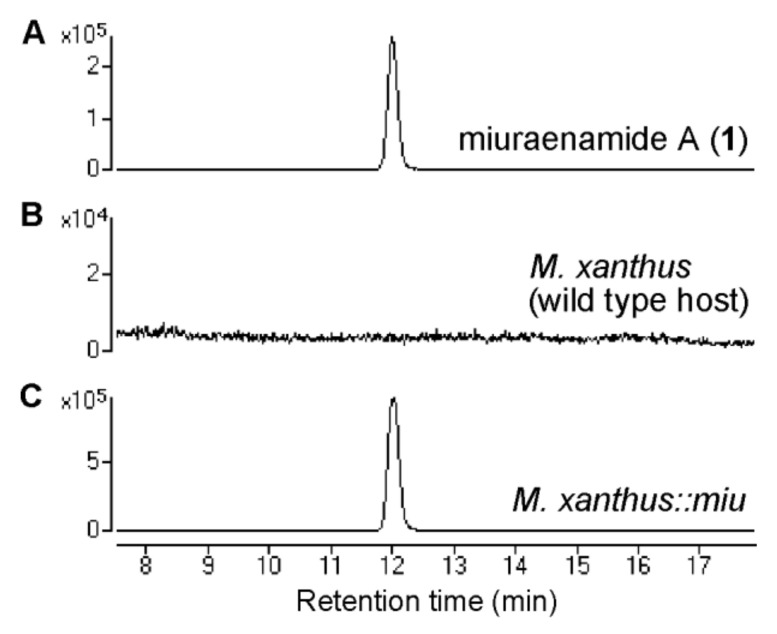
Production of miuraenamide A (**1**) using the heterologous transformant. Extracted ion chromatogram (*m/z* 684.2216) of standard **1** (**A**), an extract of the wild-type host *M. xanthus* (**B**), and an extract of the heterologous transformant harboring *miu* cluster (**C**).

There were two cutting sites for the restriction enzyme BlnI only outside the *miu* cluster. To clone the *miu* cluster, a genomic bacterial artificial chromosome (BAC) library of “*P. miuraensis*” SMH-27-4 was constructed via the complete digestion of the genome using BlnI. The BAC vector (p17-9A) that contained the full-length *miu* cluster was screened using PCR. The BAC vector was modified through the Red/ET recombination technology to replace a DNA fragment (18.5 kbp) outside the *orf29* of the *miu* cluster with “5TA-Kan”, which contained a kanamycin resistant gene and a 5.0-kbp DNA fragment homologous to a myxovirescin A biosynthetic gene *ta-1* [21] of the terrestrial myxobacterial model strain *Myxococcus xanthus*. The resulting recombinant vector *miu* BAC was integrated into the genome of *M. xanthus* via single crossover homologous recombination (Figure S1). The constructed strain *M. xanthus*::*miu* successfully produced **1**, as demonstrated using liquid chromatography-mass spectrometry (LC-MS, Figure 3), thus verifying that the *miu* cluster is responsible for the biosynthesis of **1**, although the yield was only 0.06 mg/L.

### 2.2. Proposed Mechanism for the Biosynthesis of Miuraenamide A

Since the *miu* cluster was confirmed to be the biosynthetic machinery for **1**, the function of 36 orfs comprising the cluster was predicted based on the BLASTP results obtained from the National Center for Biotechnology Information (NCBI) non-redundant protein sequences (nr) database and the minimum information on the biosynthetic gene cluster (MiBIG, version 3.1) database (Table 1). There are seven plausible structural genes designated as *miuA*–*miuG*. In addition to the backbone-constructing PKSs (MiuA, MiuB) and NRPS (MiuC) genes, the four predicted gene products were modification enzymes: cytochrome P450 (MiuD), *O*-methyltransferase (MiuE), thioesterase (MiuF), and FAD-dependent oxidoreductase/brominase (MiuG). Since the chlorinated and iodinated congeners, miuraenamides B and C, have also been isolated from “*P. miuraensis*” SMH-27-4 [22], MiuG was regarded as a halogenase.

The biosynthetic mechanism was proposed based on the organization of the *miu* cluster (Figure 2B). The first PKS (MiuA) exhibits an unusual starter module arrangement, ACP-KS-AT-AT-KR-ACP, wherein the loading domain acyltransferase (the first AT) is ripped away from the starter domain acyl carrier protein (the first ACP) by the interruption of the ketosynthase (KS) domain in the first elongation module (Module 1). This particular loading type was reported in the biosyntheses of other myxobacterial compounds such as soraphen from *Sorangium cellulosum* So ce26 [23], myxothiazol from *Stigmatella aurantiaca* DW4/3-1 [24], myxalamide from *S. aurantiaca* Sga15 [25], and chondramide from *Chondromyces crocatus* Cm c5 [26]. The first AT domain in MiuA loaded the starter molecule acetyl-CoA, and then the four polyketide elongation modules (Modules 1–4) in MiuA and MiuB added three malonyl-CoA (mal) and one methylmalonyl-CoA (mmal) in the sequence of mal-mmal-mal-mal. The resulting polyketide intermediate tethered to Module 4 in MiuB was transferred to the peptidyl carrier protein (PCP) domain carrying L-alanine by the first condensation domain (LCL) of Module 5 in MiuC. The second condensation domain (LCL) in Module 6 recruited 3-bromo-L-tyrosine (or L-tyrosine) to the alanine residue. The epimerization (E) domain of Module 6 converted the incorporated L-tyrosine to the D configuration. MiuG was predicted to be halogenase, which catalyzes the bromination of tyrosine (or of the tyrosine residue), as the closest homolog to MiuG in the MiBIG database is a marine bacterial brominase, Bmp5, which catalyzes the biosynthesis of bromophenols [27]. The DCL domain of Module 7 recruits L-phenylalanine to the C-terminus of the peptidyl unit. The incorporated L-phenylalanine is possibly converted to the D form by the epimerization domain (E) of Module 7, although the stereogenic center at C-13 will eventually be lost. MiuC, besides the three regular NRPS modules (Modules 5–7), possesses two extra domains, DCL* and phosphoenolpyruvate synthase (PEP*), whose functions are unknown. The release and cyclization of the linear polyketide/peptide from MiuC can be catalyzed via the thioesterase (TE) MiuF, yielding the tentative early intermediate **2**. The β-carbon (C-14) of phenylalanine of **2** may be oxidized by MiuD (cytochrome P450) to produce the known ketone congener miuraenamide E (**3**), which was previously isolated from the original strain SMH-27-4 as a natural congener [22]. As the final step, MiuE (*O*-methyltransferase) catalyzes *O*-methylation accompanied by the enolization of the C-14 ketone group of **3** to generate **1**.

### 2.3. Verification of Modification Enzyme Genes

**Figure 4:**
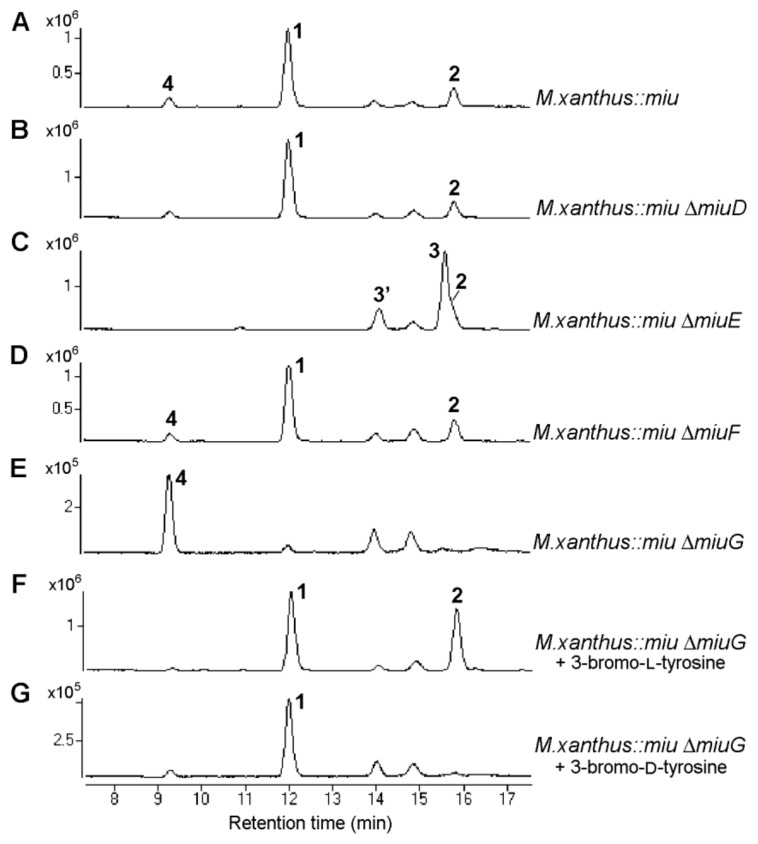
Production of miuraenamide A (**1**) and its congeners via four heterologous mutants lacking *miuD–G*. Extracted ion chromatographs (merged from *m/z* 684.2216, 606.3125, 656.2285, 670.2068, and 672.2217) of the extracts of the heterologous producer without gene disruption *M. xanthus*::*miu* (**A**), *miuD* (cytochrome P450 gene)-disrupted mutant *M. xanthus*::*miu* Δ*miuD* (**B**), *miuE* (*O*-methyltransferase gene)-disrupted mutant *M. xanthus*::*miu* Δ*miuE* (**C**), *miuF* (thioesterase gene)-disrupted mutant *M. xanthus*::*miu* Δ*miuF* (**D**), *miuG* (halogenase gene)-disrupted mutant *M. xanthus*::*miu* Δ*miuG* (**E**), *M. xanthus*::*miu* Δ*miuG* fed on 3-bromo-L-tyrosine (**F**), and *M. xanthus*::*miu* Δ*miuG* fed on 3-bromo-D-tyrosine (**G**).

To verify the proposed biosynthetic mechanism, the four estimated modification enzyme genes, *miuD*–*miuG*, were separately disrupted. The gene disruptions were conducted on the recombinant *miu* BAC using the Red/ET recombination technology (Figure S2–S5). The gene-disrupted BAC vectors were subsequently integrated into the *M. xanthus* genome via single crossover homologous recombination at the same position as that of the initially constructed transformant *M. xanthus*::*miu*, assembling the four mutants *M. xanthus*::*miu* Δ*miuD*–Δ*miuG*. The production of **1** and its congeners were detected using LC-MS (Figure 4). The mass spectra for the products are listed in Figure S10.

First, using LC-MS analysis, which can cover the molecular weights of all the expected congeners, the heterologous transformant *M. xanthus::miu* without gene disruption was demonstrated to produce not only **1** but also the related congeners **2** and **4** (Figure 4A). The congener **2** was deduced to be an early biosynthetic precursor lacking the enol ether at C-14 (Figure 2B), based on a molecular formula smaller than that of **1** by “CO” (observed difference, 27.9931; calcd., 27.9949; Figure S10). The intermediate **2** was the second major product, indicating that the following oxidation did not completely proceed in the heterologous host, whereas **2** had never been detected in the original producer SMH-27-4. The congener **4** had a molecular formula smaller than that of **1** by “^79^Br minus H” (observed difference, 77.9091; calcd., 77.9105; Figure S10), indicating that **4** was debromomiuraenamide A. These congeners **2** and **4** could be produced via incomplete reactions by the predicted enzymes oxidase (MiuD) and halogenase (MiuG), respectively. The heterologous expression may affect the activity or expression levels of certain modification enzymes. 

Although MiuD (cytochrome P450) was estimated to oxidize the phenylalanine residue of **2** to form **3**, the disruption of *miuD* did not affect the production of **1** (0.13 mg/L, Figure 4B, Table 2). Therefore, it was concluded that MiuD was not involved in the biosynthesis of **1,** and unidentified gene(s) in the host genome were responsible for the oxidation. 

The Miu E (*O*-methyltransferase) was expected to be responsible for the methylation of **3** to produce **1**. Actually, the disruption of *miuE* (mutant *M. xanthus::miu* Δ*miuE*) resulted in the abolition of the production of **1** and, instead, the accumulation of **3** (Figure 4C, Table 2), which verified the role of MiuE. The structure of **3** was determined by comparing the LC-MS data with the known product miuraenamide E, which was previously isolated from the original producer SMH-27-4 [22]. The compound **3′** was estimated as 13-*epi*-**3**, based on the same molecular formula and susceptibility of the position 13 of **3** for racemization. Another related product **2** was also observed at a similar retention time to that of **3**. The accumulation of **2** was also observed in other heterologous mutants (Figure 4A,B,D). The absence of **4** is probably due to the lack of *O*-methylation of the corresponding debromo-precursor. 

MiuF (thioesterase) was proposed to release and cyclize the precursor polyketide/peptide chain. However, the disruption of this gene did not affect the production of **1** (0.08 mg/L, Figure 4D, Table 2). This suggests that there is another responsible protein, probably the extra DCL* (function-unassigned DCL) domain in MiuC, because this condensation domain contains the HHXXXDX_14_Y motif, the conserved motif of the condensation enzyme SgcC5 for ester bond formation [28]. 

MiuG (halogenase) was suggested to be responsible for the bromination of tyrosine. The knockout of *miuG* (*M. xanthus::miu* Δ*miuG*) resulted in the abolition of the production of **1** and, instead, the accumulation of **4** (Figure 4E and Figure S10), verifying that MiuG is the bromination enzyme for tyrosine. As this disruption mutant fed on 3-bromo-L-tyrosine, it entirely restored the production of **1** (0.09 mg/L, Figure 4F, Table 2); the condensation domain LCL in Module 6 highly preferred bromotyrosine over tyrosine and MiuG probably preferred free tyrosine over the tyrosine residue on the peptide chain. Feeding the mutant on 3-bromo-D-tyrosine also restored the production of **1**, although the yield was lower (0.02 mg/L, Figure 4G, Table 2) than that for the L-form. Similar results were observed for the original transformant *M. xanthus::miu* (0.16 mg/L for L-form and 0.07 mg/L for D-form, Figure S11), supporting the preference of the LCL domain in Module 6 for L-amino acid. As the enantiomeric purity of 3-bromo-D-tyrosine used in this study was quite high (99.7% ee), it may be utilized after being converted to the L-form by an amino acid racemase of the host bacterium, although its incorporation efficiency was lower than that of the L-form (Table 2), which was the case with *M. xanthus::miu* (Figure S11). 

### 2.4. Verification of Unknown Genes Using Multigene Disruption

**Figure 5:**
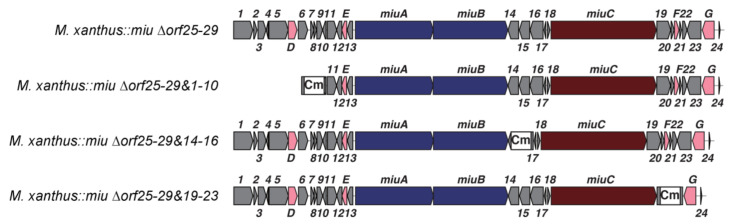
Gene organizations of *miu* clusters lacking multigene regions. Cm represents the chloramphenicol resistance gene that was used as a selection marker.

**Figure 6:**
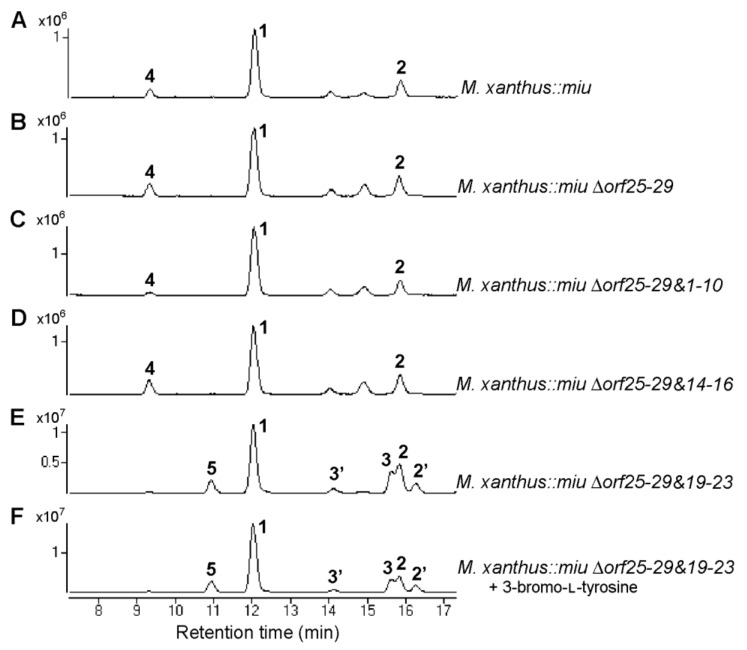
Production of miuraenamide A (**1**) and its congeners using multigene-disrupted heterologous hosts. Extracted ion chromatogram (merged from *m/z* 684.2216, 606.3125, 656.2285, 670.2068, and 672.2217) of the extracts of *M. xanthus*::*miu* (**A**), *M. xanthus*::*miu ∆orf25–29* (**B**), *M. xanthus*::*miu* Δ*orf25–29&1–10* (**C**), *M. xanthus*::*miu* Δ*orf25–29&14–16* (**D**), and *M. xanthus*::*miu* Δ*orf25–29&19–23* (**E**,**F**).

The gene disruption experiments revealed that two modification enzyme genes (*miuE* and *miuG*) were involved in the biosynthesis of **1** in addition to the core genes *miuA–C*. The other function-unknown genes were clustered into the following four regions, *orf1–10*, *orf14–16*, *orf19–23*, and *orf25–29* (Figure 2A). These regions were next removed to explore whether they were related to the biosynthesis of **1** (Figure 5 and Figure S6–S9 from Supplementary Materials). As there are apparent non-coding regions between *miuG* and *orf25* (Figure 2A), it is likely that the region *orf25–29* is not involved in the biosynthesis of **1**. This region was removed from the above-mentioned BAC vector p17-9A using the Red/ET recombination technology, as this region was located at the end of the *miu* cluster (Figure 2A). The recombinant *miu* BAC ∆*orf25–29* was subsequently integrated into the *M. xanthus* genome via single crossover homologous recombination. The mutant *M. xanthus::miu* ∆*orf25–29* produced **1** in a yield of 0.07 mg/L, similar to that of *M. xanthus::miu* (Figure 6B, Table 2 and Table 3), indicating that the real BGC ranged between *orf1* and *orf24* (Figure 5).

**Figure 7:**
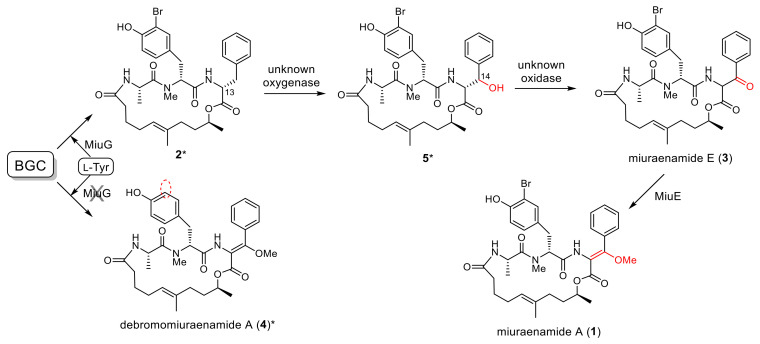
Plausible biosynthetic route for miuraenamide A (**1**) and related congeners. The compounds with an asterisk were estimated by the molecular formulae obtained using high-resolution MS.

The subsequent disruptions of the other regions were, therefore, performed on this mutant *M. xanthus::miu* Δ*orf25–29*. The two double-disruption mutants *M. xanthus::miu* Δ*orf25–29&1–10* and *M. xanthus::miu* Δ*orf25–29&14–16* also normally produced **1** in yields of 0.07–0.10 mg/L (Figure 6C,D, Table 3). The result of the former mutant indicates that the minimal range of the BGC is *orf11–24* (62.1 kbp) or narrower. On the other hand, following the removal of the region *orf19–23*, the resulting mutant *M. xanthus::miu* Δ*orf25–29&19–23* produced **1** in the highest yield of 0.70 mg/L (Figure 6E, Table 3). In addition, the metabolic profile of this mutant was complicated, namely the production of several related metabolites **3**, **2′**, **3′**, and **5**. The products **2′** and **3′** were estimated as the C-13 epimers of **2** and **3**, respectively, because of their same molecular formulae and similar retention times (Figure 6E). Compound **5** possessed a molecular formula larger than that of **2** by oxygen atom (observed difference, 15.9932; calcd., 15.9949; Figure S10), which corresponds to a hydroxylated **2**. We proposed a plausible structure for **5** (Figure 7), wherein the phenylalanine residue is hydroxylated at C-14. The region *orf19–23* may contain factor(s) affecting the transcription of some genes constituting the *miu* cluster. Interestingly, the feeding of the mutant *M. xanthus::miu* Δ*orf25–29&19–23* on 3-bromo-L-tyrosine boosted the yield of **1** to 1.21 mg/L (Table 3), which corresponded to a 20-fold increase compared with that of the original transformant *M. xanthus::miu* (Table 2), and was slightly higher than that of the original producer SMH-27-4. 

## 3. Discussion

The antifungal and antitumor antibiotic miuraenamide A (**1**) is produced by the slightly halophilic myxobacterium “*P. miuraensis*” SMH-27-4. As this strain is a hard-to-culture rare marine myxobacteria, genetic engineering of the biosynthetic machinery is essential to the effective production of the valuable antibiotic **1**. The BGC for **1** (*miu* cluster) was successfully cloned and heterologously expressed in the well-known terrestrial myxobacterium *M. xanthus*. Although the obtained heterologous transformant *M. xanthus::miu* was considerably easier to treat and grew faster than the original strain SMH-27-4, the productivity of **1** was quite low (6% of that of the original strain). The proposed biosynthetic mechanism of the *miu* cluster was partially verified via gene disruption experiments using the transformant *M. xanthus::miu*. The type I PKSs (MiuA and MiuB) and NRPS (MiuC) recruit and sequentially couple C_2_/C_3_ carboxylic acid and amino acid units to generate the early intermediate **2** (Figure 7). The thioesterase gene *miuF*, although regarded as a candidate gene responsible for the release and cyclization of the enzyme-bound linear precursor, was found not to be involved in the biosynthesis of **1**. Instead, this reaction may be catalyzed by the DCL* domain in MiuC because it shares the conserved motif for ester-bond formation with the condensation enzyme SgcC5 [28]. Therefore, in the biosynthesis of **1**, the DCL* domain may catalyze the formation of the ester bond between the hydroxy group of C-9 and the carbonyl group of D-phenylalanine, leading to the release and cyclization of the polyketide/peptide chain. As this domain is D-specific for the peptidyl donor, the configuration at C-13 of the phenylalanine residue in **2** is possibly *R* (D), as indicated in Figure 7. The detection of **2**, **3**, and **5** in this study suggested the presence of an oxidation enzyme gene that catalyzes oxygen transfer to the β-carbon (C-14) of phenylalanine. Although *miuD* encoding cytochrome P450 appeared to be the exclusive candidate for this reaction within the *miu* cluster, the *miuD*-disrupted mutant still produced **1**. Although we searched for oxidation-related functions in the untrimmed orfs (*orf11–13*, *orf17–18*), any meaningful functions could not be found. The fact that no other orfs in the *miu* cluster were annotated as oxidation enzymes indicated the presence of a responsible oxygenase outside the cluster. The *O*-methyltransferase MiuE was readily confirmed as being responsible for the methylation of **3** to the final product **1** through gene disruption. The halogenase MiuG was found to be the tyrosine bromination enzyme that utilized free L-tyrosine as the substrate because MiuG inactivation generated the unnatural congener debromomiuraenamide A (**4**), and the production of **1** was restored by feeding on bromotyrosine. The A domain of Module 6 seems to prefer 3-bromo-L-tyrosine over tyrosine. Although 3-bromo-L-tyrosine seemed to be useful for the better production of **1**, the feeding experiments with this biosynthetic precursor never worked on the *miuG*-disrupted mutant as expected. However, the multigene deletion experiments created a breakthrough leading to a drastic increase in the yield of **1** and the construction of more compact *miu* clusters. The deletion of 15 orfs in total (*orf25*–*29* and *orf1*–*10*) did not affect the production of **1**, indicating that the remaining 20 orfs extending over 62.1 kbp (corresponding to 72% of the original *miu* cluster) were adequate for the biosynthesis of **1** (Figure 5). On the other hand, the removal of the *orf25*–*29* and *orf19*–*23* regions substantially increased the yield of **1** and resulted in a complicated metabolic profile (Figure 6E), suggesting the presence of unknown gene(s) in the *orf19–23* region that affects transcriptional regulation of some of the functionally defined *miu* genes or some alteration of the *miu* gene expression level by removing this unnecessary region. Despite a careful search for the functions of *orf19–23*, any plausible functions (e.g., DNA binding domain of a repressor) could not be found. Interestingly, feeding this mutant *M. xanthus::miu* Δ*orf25*–*29&19*–*23* on 3-bromo-L-tyrosine promoted the production of **1** at a slightly higher level (1.2 mg/L) than the original SMH-27-4 strain (1 mg/L). Considering the growth rate of this mutant (4 days), the production efficiency is five times higher than that of SMH-27-4 (18 days of culture). Further studies are needed to unveil the function of unidentified genes in the *miu* cluster for a much more effective heterologous production of **1**. 

## 4. Materials and Methods

### 4.1. Bacterial Strains and Culture Conditions

All bacterial strains, plasmids, and primers (Eurofins Genomics, Tokyo, Japan) used in this study are listed in Table S1–S3. The myxobacterium “*P. miuraensis*” SMH-27-4 was cultivated as previously described [11]. CTT medium [29] was used for the preculture of *M. xanthus* ATCC 25232 (wild type) and its mutants at 30 °C. PM1 medium (see below) supplemented with 2% (*w*/*v*) Sepabeads SP207 resin (Mitsubishi Chemical Co., Tokyo, Japan) was used for the heterologous production of **1**. 

PM1 medium: 1% (*w*/*v*) Bacto™ casitone (Thermo Fisher Scientific, Waltham, MA, USA), 1% (*w*/*v*) HEPES, 0.2% (*w*/*v*) Bacto™ malt extract (Thermo Fisher Scientific), 0.1% (*w*/*v*) Bacto™ yeast extract (Thermo Fisher Scientific) and 0.18% (*w*/*v*) MgSO_4_·7H_2_O. pH was adjusted to 7.0 with 1 M NaOH before autoclaving. A vitamin B_12_ solution was sterilized via filtration and added to the autoclaved broths at the final concentration of 0.1 mg/L.

### 4.2. Chemicals

The antibiotics chloramphenicol and kanamycin were purchased from FUJIFILM Wako Pure Chemical Co. (Osaka, Japan). They were added to media at the final concentrations of 35 µg/mL and 50 µg/mL, respectively. 

The 3-Bromo-L- and D-tyrosines were synthesized according to the published protocol [30] except for the purification method. The crude product was purified using HPLC under the following conditions: a Develosil ODS-HG-5 column (i. d. 20 × 250 mm) (Nomura Chemical, Aichi, Japan), a solvent program of 15–50% (35 min) acetonitrile in water, 0.1% trifluoroacetic acid (TFA), and a flow rate of 8 mL/min. The fraction containing 3-bromotyrosine was concentrated and freeze-dried to give TFA salt as colorless needles. The yields from 900 mg of L- and D-tyrosines were 362 mg and 345 mg, respectively. 3-Bromo-L-tyrosine: [α]^15^_D_ –3.0 (c 0.50, 1.0 M HCl), 3-bromo-D-tyrosine: [α]^15^_D_ +3.7 (c 0.46, 1.0 M HCl). Filter-sterilized bromotyrosine solutions were added to autoclaved broths at the final concentration of 0.5 mM. 

The enantiomeric purity of 3-bromo-L- and D-tyrosines was determined by Marfey’s method at 99.3% and 99.7%, respectively (Figure S12). Briefly, a mixture of 3-bromotyrosine (TFA salt, 0.3 mg), water (30 μL), 1 M NaHCO_3_ (20 μL), and 1% *N*^α^-(5-fluoro-2,4-dinitrophenyl)-L-alaninamide (L-FDAA) (TCI, Tokyo, Japan) in acetone (30 μL) was kept at 37 °C for 1 h. The mixture was neutralized with 1 M HCl (20 μL) and diluted with MeCN (100 μL), and a portion (3 μL) was analyzed using HPLC (Develosil ODS-UG-5 (i. d. 4.6 × 250 mm), 40% MeCN in 0.1% TFA, 1 mL/min, detected at 320 nm). 3-Bromo-L- and D-tyrosines were eluted at 10.9 and 12.3 min, respectively.

### 4.3. PCR and Products Purification

The prepared transformants were verified by PCR experiments using GoTaq^®^ Green Master Mix (Promega, Madison, WI, USA) and primer pairs listed in Table S3 under the following conditions: pre-denature at 94 °C for 5 min; 40 cycles of denature at 94 °C for 30 s, annealing at 53 °C for 30 s, and extension at 72 °C for 1 min/kbp; and final extension at 72 °C for 7 min. The DNA fragments used for cloning were amplified by PCR using the enzyme KOD FX Neo (TOYOBO, Osaka, Japan) under the following conditions: pre-denature (94 °C, 2 min); 45 cycles of denature (98 °C, 10 s), and extension (68 °C, 60 s/kbp). 

### 4.4. Construction and Screening of a Genomic BAC Library

The *miu* cluster was identified via antiSMASH analysis of the draft genome of “P. miuraensis” SMH-27-4 (GenBank accession number: JAOVZF000000000.1) [20]. The genomic DNA was isolated following the protocols described previously [20]. Seven µg of genomic DNA was completely digested with 40 U BlnI (Takara Bio, Kusatsu, Shiga, Japan) overnight at 37 °C, and then separated on 0.5% Certified™ Low Melt Agarose gel (Bio-Rad Laboratories, Hercules, CA, USA) via gel electrophoresis. DNA fragments around 50–165 kb in size were extracted from the agarose gel using Thermostable β-Agarase (NIPPON GENE Co., Toyama, Japan). The DNA was purified from the digested solution via ethanol precipitation [31]. The bacterial artificial chromosome (BAC) vector pCC1BAC-BlnI was derived from the commercial pCC1BAC (Epicentre Biotechnologies, WI, USA). The pCC1BAC was linearized by PCR using the primer pair pCC-BlnI 2 F/R and introducing BlnI cutting sites at both ends. Following electrophoresis, the PCR product was purified from agarose gel using Wizard SV Gel and the PCR Clean-Up System (Promega). The purified DNA was digested with BlnI for 2 h, and then washed with an equivalent amount of PCI solution (phenol:chloroform:isoamyl alcohol, 25:24:1). Following ethanol precipitation and redissolved in Milli-Q water, the BlnI digested pCC1BAC-BlnI was treated with alkaline phosphatase CIAP (Takara Bio) and ligated with the purified 50–165 kb genomic DNA fragments using a Takara DNA Ligation Kit Long (Takara Bio). The ligation product was transformed into *E. coli* HST08 premium Electro-Cells (Takara Bio) to generate a genomic BAC library consisting of 1920 clones. The primer pair miuBGC p F/R located at the second KS domain of *miuB* was used for screening the genomic library. The recombinant BAC p17-9A clone harboring the complete *miu* cluster was verified through PCR experiments using the primer pairs T1PKS1 cF/R, NPRS cF/R, and FMO cF/R located in *miuA*, *miuC*, and *miuG*, respectively. 

### 4.5. Construction of Red/ET Recombination Modification Cassette 5TA-Kan^R^

The genomic DNA of *M. xanthus* was isolated with the QIAamp DNA Mini Kit (Qiagen, Hilden, Germany). The 5TA fragment was amplified from the myxovirescin A biosynthetic gene *ta-1* of *M. xanthus* with the primer pair loTA Gib F/R. The vector pTA-Kan^R^ [32] was linearized by PCR using the primer pair ploTA F/R. The PCR products were purified via electrophoresis on 1% agarose gel followed by extraction from the gel using a FavorPrep GEL/PCR Purification Mini Kit (Favorgen Biotech Corp., Taiwan, China). The concentration of the DNA solutions was measured on a V-730BIO UV/visible spectrophotometer (JASCO, Tokyo, Japan). The DNA fragment “5TA” was assembled to the linearized vector using NEBuilder^®^ HiFi DNA Assembly Master Mix (NEW ENGLAND BioLabs Inc., Ipswich, MA, USA) at the DNA molar ratio of 3:1 (0.2 pmol in total) to generate the plasmid p5TA-Kan^R^ according to the manufacturer’s manual. A portion (2 µL) of the assembled product was transformed into Competent high DH5α (TOYOBO) according to the manufacturer’s manual. The resulting colonies were verified by PCR using the primer pair ploTA check F/R located at one end of the assembly site. The Red/ET recombination modification cassette 5TA-Kan^R^ was amplified by PCR using the assembled plasmid p5TA-Kan^R^ as the template.

### 4.6. Modification of BAC Vector to miu BAC via Red/ET Rcombination

The BAC vector p17-9A containing the *miu* cluster was purified using the QIAGEN Plasmid Midi Kit (Qiagen). Approximately 200 ng of the BAC p17-9A was transformed into 70 µL of electrocompetent *E. coli* SW105 cells [33] in a 1-mm cuvette (NEPAGENE, Ichikawa, Chiba, Japan) at 1,800 V for one pulse. *E.coli* transformants were recovered at 30 °C for 1 h in 1 mL of SOC medium (Takara Bio) and then plated onto LB medium (Thermo Fisher Scientific) containing chloramphenicol at 30 °C for 24–36 h. Individual colonies were picked up and verified by colony PCR. 

The Red/ET recombination modification cassette 5TA-Kan^R^ was amplified using the primer pair ploTA-Kan red F/R from the vector p5TA-Kan^R^. Approximately 200 ng of the modification cassette “5TA-Kan^R^” was transformed into electrocompetent *E. coli* SW105 cells harboring the BAC vector p17-9A via electroporation followed by recovery and cultivation under the above-mentioned conditions to obtain the recombinant *miu* BAC harboring the complete *miu* cluster and 5.0 kbp *M. xanthus* homologous fragment (5TA). The resulting colonies were verified by PCR using the two primer pairs loTA cF/R and kanf/pCC1BAC R (Figure S1). 

### 4.7. Gene Disruption

The disruption of the modification genes *miuD–miuG* was performed on the *miu* BAC (Figures S2–S5). The chloramphenicol gene fragment “Cm” was amplified using the template pCC1BAC and the primer pairs Cm-miuD-F/R, Cm-miuE-F/R, Cm-miuF-F/R, and Cm-miuG-F/R for the disruption of *miuD-miuG*, respectively. The PCR products were purified by the above-mentioned methods. Approximately 200 ng of “Cm” was transformed into electrocompetent *E. coli* SW105 cells harboring *miu* BAC via electroporation followed by recovery and cultivation under the above-mentioned conditions to obtain the four gene-disrupted BAC vectors. 

The removal of a terminal region (*orf25–29*) of the *miu* cluster was performed on the BAC vector p17-9A (Figure S6). The gene cassette 5TA-Kan^R^ was amplified using the primer pair 5TA-orf25-F/ploTA-Kan red R from the vector p5TA-Kan^R^. The PCR product was purified by the above-mentioned method. Approximately 200 ng of “5TA-Kan” was transformed into electrocompetent *E. coli* SW105 cells harboring p17-9A via electroporation followed by recovery and cultivation under the above-mentioned conditions to obtain *miu* BAC ∆*orf25–29*.

The removal of the other three gene regions (*orf1–10*, *orf14–16*, *orf19–23*) were performed on *miu* BAC ∆*orf25–29* (Figures S7–S9). The chloramphenicol gene fragment “Cm” was amplified using the template pCC1BAC and the primer pairs Cm-orf1-F/Cm-orf10-R, Cm-orf14-F/Cm-orf16-R, and Cm-orf19-F/Cm-orf23-R for the disruption of the gene regions *orf1–10*, *orf14–16*, and *orf19–23*, respectively. The PCR products were purified by the above-mentioned methods. Approximately 200 ng of each “Cm” was transformed into electrocompetent *E. coli* SW105 cells harboring *miu* BAC ∆*orf25–29* via electroporation followed by recovery and cultivation under the above-mentioned conditions to obtain the three BAC vectors: *miu* BAC ∆*orf25–29&1–10*, *miu* BAC ∆*orf25–29&14–16*, and *miu* BAC ∆*orf25–29&orf19–23*. 

### 4.8. Construction of M. xanthus Transformants

Approximately 10 µg of the recombinant *miu* BAC harboring the complete *miu* cluster and the 5.0 kbp *M. xanthus* homologous fragment was transformed into 240 µL electrocompetent *M. xanthus* cells in a 2-mm cuvette (NEPAGENE) at 1,500 V, 5 ms, two pulses. For the preparation of the electrocompetent cells, *M. xanthus* were harvested when OD_600_ reached 0.2–0.6 in CTT medium. After precooling on ice for 20 min, the cells were washed with ice-cold Milli-Q water three times and dissolved in Milli-Q water of 1/100 volume of the culture medium. *M. xanthus* transformants were recovered at 30 °C for 10–12 h in 3 mL of CTT medium and then plated onto CTT medium containing kanamycin. The resulting colonies that appeared after 5–7 days were verified by PCR experiments using the four primer pairs T1PKS1 cF/R, T1PKS2 cF/R, loupF/R, 0lodownF/2lodownR located at *miuA*, *miuB*, and two integration sites (Figure S1). Other *M. xanthus* transformants were constructed via the same method.

### 4.9. Production of Miuraenamide A (**1**) Using M. xanthus Heterologous Mutants

The *M. xanthus* mutants were cultivated in 50 mL of the PM1 medium supplemented with 2% (w/v) Sepabeads SP207 absorber resin. The cells and resin were harvested after 4 days of culture via centrifugation and extracted twice with acetone (30 mL) by shaking at 30 °C for 30 min. After filtration, the combined filtrates were concentrated in vacuo and dried to produce a yellow oil. The extract was dissolved in 70% MeOH (5 mL), and a portion (5 µL) equivalent to 0.05 mL broth was subjected to LC-MS analysis. 

### 4.10. LC-MS Analysis of Miuraenamide A (**1**) and Congeners (**2**–**5**)

HPLC was performed using an Agilent 1100 HPLC system (Agilent Technologies, Santa Clara, CA) under the following conditions: a Cadenza CD-C18 column (i. d. 3 × 50 mm, Imtakt, Kyoto, Japan), a solvent program of 40–75% (20 min) acetonitrile in water, and a flow rate of 0.2 mL/min. MS coupled to the HPLC system was performed on an Agilent 6520 Accurate-Mass Q-TOF spectrometer in the mass range of *m/z* 50–1700 in positive ion mode. For the quantitative analysis of **1**, 0.1 and 0.5 µM solutions of the standard **1** were used. The congeners **2**–**5** were not quantified due to the lack of their standard samples. **1** (*t*_R_ = 12.0 min): *m/z* 684.2216 (calcd for C_34_H_43_^79^BrN_3_O_7_, 684.2279) and 706.2032 (calcd for C_34_H_42_^79^BrN_3_O_7_Na, 706.2098); **2** (*t*_R_ = 15.8 and 16.2 min): *m/z* 656.2285 (calcd for C_33_H_43_^79^BrN_3_O_6_, 656.2330) and 678.2100 (calcd for C_33_H_42_^79^BrN_3_O_6_Na, 678.2149); **3** (*t*_R_ = 15.6 and 14.1 min): *m/z* 670.2068 (calcd for C_33_H_41_^79^BrN_3_O_7_, 670.2122) and 692.2055 (calcd for C_33_H_40_^79^BrN_3_O_7_Na, 692.1942); **4** (*t*_R_ = 9.3 min): *m/z* 606.3125 (calcd for C_34_H_44_N_3_O_7_, 606.3174) and 628.2941 (calcd for C_34_H_43_N_3_O_7_Na, 628.2993); **5** (*t*_R_ = 10.9 min): 672.2217 (calcd for C_33_H_43_^79^BrN_3_O_7_, 627.2279) and 694.2038 (calcd for C_33_H_42_^79^BrN_3_O_7_Na, 694.2098).

## Figures and Tables

**Table 1:** Predicted functions of the orfs in the *miu* cluster-containing region (Figure 2A).

Orfs	Size (aa)	Closest Homolog (BLASTP)	Origin	Accession Number	Identity/Similarity (%)
*orf1*	1008	Hypothetical protein	*Nannocystis pusilla*	WP_224196465.1	40/53
*orf2*	173	Hypothetical protein	*Nannocystis exedens*	WP_096327445.1	29/40
*orf3*	418	Hypothetical protein	Myxococcales bacterium	MCA9650222.1	75/86
*orf4*	104	Polyhydroxyalkanoic acid system family protein	Deltaproteobacteria bacterium	MCH9682166.1	73/86
*orf5*	1020	Spermidine synthase	Deltaproteobacteria bacterium	MCH9682165.1	81/88
*miuD*	461	Cytochrome P450	Myxococcales bacterium	MCB9753755.1	67/77
*orf6*	493	Peptidase M4 family protein	Deltaproteobacteria bacterium	RME25565.1	51/65
*orf7*	171	Hypothetical protein	*Balneola* sp.	MBE78502.1	27/49
*orf8*	138	DUF4398 domain-containing protein	bacterium	MCR9159996.1	46/67
*orf9*	302	OmpA family protein	Deltaproteobacteria bacterium	MBC8068113.1	47/63
*orf10*	182	Hypothetical protein	Deltaproteobacteria bacterium	MCH9682164.1	69/80
*orf11*	497	OmpA family protein	Myxococcales bacterium	MCA9650212.1	69/84
*orf12*	274	FHA domain-containing protein	Myxococcales bacterium	MCA9652678.1	58/68
*miuE*	257	O-methyltransferase	*Stigmatella erecta*	SEU19554.1	35/54
*orf13*	288	Lysine-specific demethylase 8 isoform X1	*Micropterus salmoides*	XP_038559593.1	31/47
*miuA*	4105	Type I polyketide synthase	*Pyxidicoccus fallax*	WP_169347329.1	60/72
*miuB*	3926	Amino acid adenylation domain-containing protein	*Pyxidicoccus fallax*	NPC81269.1	51/64
*orf14*	554	Mechanosensitive ion channel family protein	*Vitiosangium* sp. GDMCC 1.1324	WP_108076111.1	40/62
*orf15*	545	Hemopexin repeat-containing protein	*Nannocystis* sp. fl3	WP_269038991.1	75/87
*orf16*	686	Heavy metal translocating P-type ATPase	Proteobacteria bacterium	MBU0970734.1	41/63
*orf17*	94	Hypothetical protein	Myxococcales bacterium	MBL8970963.1	60/76
*orf18*	125	Hypothetical protein	Myxococcales bacterium	MBL8970963.1	70/83
*miuC*	5546	Non-ribosomal peptide synthetase	*Chondromyces crocatus*	WP_169796632.1	40/55
*orf19*	730	Bifunctional metallophosphatase/5′-nucleotidase	*Chondromyces crocatus*	WP_050432501.1	59/74
*orf20*	164	GTPase	*Candidatus* Methylumidiphilus alinenensis	PZN75038.1	49/71
*miuF*	241	Thioesterase	Myxococcales bacterium	MCA9716988.1	62/75
*orf21*	95	Hypothetical protein	Myxococcales bacterium	MBL8970963.1	67/81
*orf22*	233	PEP/pyruvate-binding domain-containing protein	Myxococcales bacterium	MBL8970530.1	62/72
*orf23*	728	Heavy metal translocating P-type ATPase	*Nannocystis* sp. MB1016	ALD82534.1	61/79
*miuG*	621	FAD-dependent oxidoreductase;	*Symploca* sp. SIO1A3;	NER47269.1	39/54
		Bmp5, Flavin-dependent single-component *p*-hydroxybenzoate brominase/decarboxylase (from MIBiG database)	*Pseudoalteromonas phenolica* O-BC30	KF540211.1	35/52
*orf24*	58	No significant homology			
*orf25*	235	OmpA family protein	Myxococcales bacterium	MCA9650211.1	61/79
*orf26*	771	LysM peptidoglycan-binding domain-containing protein	Myxococcales bacterium	MCA9705957.1	66/78
*orf27*	441	Protein kinase	Deltaproteobacteria bacterium	MCH9682160.1	69/80
*orf28*	599	HAMP domain-containing protein	Myxococcales bacterium	MCA9650207.1	76/86
*orf29*	594	PAS domain S-box protein	Myxococcales bacterium	MCA9650206.1	66/76

**Table 2:** Production of miuraenamide A (**1**) and its congeners in heterologous mutants lacking *miuD–G*.

Heterologous Host	Yield of 1 (mg/L)	Produced Congeners
*M. xanthus*::*miu*	0.06	**2, 4**
*M. xanthus*::*miu* Δ*miuD*	0.13	**2, 4**
*M. xanthus*::*miu* Δ*miuE*	-	**2, 3, 3′**
*M. xanthus*::*miu* Δ*miuF*	0.08	**2, 4**
*M. xanthus*::*miu* Δ*miuG*	-	**4**
*M. xanthus*::*miu* Δ*miuG* + 3-bromo-L-tyrosine	0.09	**2**
*M. xanthus*::*miu* Δ*miuG* + 3-bromo-D-tyrosine	0.02	**4**

**Table 3:** Production of miuraenamide A (**1**) and its congeners in multigene-disrupted heterologous mutants.

Heterologous Host	Yield of 1 (mg/L)	Produced Congeners
*M. xanthus*::*miu* ∆*orf25–29* (31 orfs, 77.0 kbp)	0.07	**2, 4**
*M. xanthus*::*miu* ∆*orf25–29&1–10* (20 orfs, 62.1 kbp)	0.10	**2, 4**
*M. xanthus*::*miu* ∆*orf25–29&14–16* (28 orfs, 72.6 kbp)	0.07	**2, 4**
*M. xanthus*::*miu* ∆*orf25–29&19–23* (25 orfs, 70.9 kbp)	0.70	**2, 2′, 3, 3′, 5**
*M. xanthus*::*miu* ∆*orf25–29&19–23* + 3-bromo-L-tyrosine	1.21	**2, 2′, 3, 3′, 5**

## Data Availability

The data presented in this study are available in the Supplementary Materials and via accession numbers described in Section 4 of this article.

## Supplementary Materials

The following supporting information can be downloaded at: https://www.mdpi.com/article/10.3390/molecules28062815/s1, Table S1: Bacterial strains used in this study; Table S2: Plasmids used in this study; Table S3: Primers used in this study; Figure S1: Construction of M. xanthus::miu; Figure S2: Construction of M. xanthus::miu ∆miuD; Figure S3: Construction of M. xanthus::miu ∆miuE; Figure S4: Construction of M. xanthus::miu ∆miuF; Figure S5: Construction of M. xanthus::miu ∆miuG; Figure S6: Construction of M. xanthus::miu ∆orf25–29; Figure S7: Construction of M. xanthus::miu ∆orf25–29&1–10; Figure S8: Construction of M. xanthus::miu ∆orf25–29&14–16; Figure S9: Construction of M. xanthus::miu ∆orf25–29&19–23; Figure S10: Mass spectra of 1 and its congeners. Figure S11: Production of miuraenamide A (1) and its congeners via M. xanthus::miu mutant fed on bromotyrosine. Figure S12: Enantiomeric excess of 3-bromo-D- and L-tyrosines determined through Marfey’s method.
