# Supplementary material for: Heterologous Biosynthesis of Myxobacterial Antibiotic Miuraenamide A
Source: Molecules. 2023 Mar 20;28(6):2815. doi: 10.3390/molecules28062815 (PMC10051162; doi:10.3390/molecules28062815)
Supplement: Supplementary file 1 [file molecules-28-02815-s001.zip › molecules-2253265-supplementary.pdf]

molecules

MDPI

*Supplementary Materials*

# Heterologous Biosynthesis of Myxobacterial Antibiotic Miuraenamide A

**Ying Liu, Satoshi Yamazaki and Makoto Ojika ***

Department of Applied Biosciences, Graduate School of Bioagricultural Sciences, Nagoya University, Nagoya 464-8601, Japan
* Correspondence: ojika@agr.nagoya-u.ac.jp

Contents

**Table S1**. Bacterial strains used in this study.
**Table S2**. Plasmids used in this study.
**Table S3.** Primers used in this study.
**Figure S1.** Construction of *M. xanthus*::*miu*.
**Figure S2.** Construction of *M. xanthus*::*miu* Δ*miuD*.
**Figure S3.** Construction of *M. xanthus*::*miu* Δ*miuE*.
**Figure S4.** Construction of *M. xanthus*::*miu* Δ*miuF*.
**Figure S5**. Construction of *M. xanthus*::*miu* Δ*miuG*.
**Figure S6.** Construction of *M. xanthus*::*miu* Δ*orf25-29*.
**Figure S7.** Construction of *M. xanthus*::*miu* Δ*orf25-29&1-10*.
**Figure S8.** Construction of *M. xanthus*::*miu* Δ*orf25-29&14-16*.
**Figure S9**. Construction of *M. xanthus*::*miu* Δ*orf25-29&19-23*.
**Figure S10**. Mass spectra of miuraenamide A and its congeners.
**Figure S11.** Production of miuraenamide A (**1**) and its congeners by *M.xanthus*::*miu* mutant fed on bromotyrosine.
**Figure S12**. Enantiomeric excess of 3-bromo-D and L-tyrosines determined by Marfey's method.

**Table S1.** Bacterial strains used in this study.

| Strain | Description |
| --- | --- |
| *"P. miuraensis"* SMH-27-4 | Original miuraenamide A producer (Ajinomoto) |
| *E.coli* HST08 premium | Cloning host for constructing genomic library (Takara bio) |
| *E.coli* DH5$\alpha$ | General cloning host (Toyobo) |
| *E.coli* SW105 | Red/ET recombineering host (NCI) |
| *M. xanthus* ATCC 25232 | Wild type, heterologous expression host (Ajinomoto) |
| *M. xanthus*::*miu* | Heterologous transformant expressing miuraenamide A biosynthetic gene cluster (*miu* cluster) |
| *M. xanthus*::*miu* Δ*miuD* | *miu*D gene-disrupted *M. xanthus*::*miu* |
| *M. xanthus*::*miu* Δ*miuE* | *miuE* gene-disrupted *M. xanthus*::*miu* |
| *M. xanthus*::*miu* Δ*miuF* | *miu*F gene-disrupted *M. xanthus*::*miu* |
| *M. xanthus*::*miu* Δ*miuG* | *miu*G gene-disrupted *M. xanthus*::*miu* |
| *M. xanthus::miu* Δ *orf25-29* | *orf25-29* genes-disrupted *M. xanthus*::*miu* |
| *M. xanthus*::*miu* Δ*orf25-29&1-10* | *orf25-29* and *orf1-10* genes-disrupted *M. xanthus*::*miu* |
| *M. xanthus*::*miu* Δ*orf25-29&14-16* | *orf25-29* and *orf14-16* genes-disrupted *M. xanthus*::*miu* |
| *M. xanthus*::*miu* Δ*orf25-29&19-23* | *orf25-29* and *orf19-23* genes-disrupted *M. xanthus*::*miu* |

**Table S2.** Plasmids used in this study.

| Plasmid | Description |
| --- | --- |
| pCCIBAC | BAC cloning vector, $Chl^R$ (Epicentre) |
| p17-9A | pCCIBAC-derived, harbouring *miu* cluster, $Chl^R$ |
| pTA-$Kan^R$ | pGEM-T easy vector-derived, harbouring 1.8 kb *M. xanthus* homologous fragment, $Amp^R$, $Kan^R$ [31] |
| p5TA-$Kan^R$ | pTA-$Kan^R$-derived, harbouring 5 kb *M. xanthus* homologous fragment, $Amp^R$, $Kan^R$ |
| *miu* BAC | p17-9A-derived, harbouring *miu* cluster, 5TA, $Kan^R$ |
| *miu* BAC Δ*miuD* | *miu* BAC-derived, *miuD* gene was disrupted, $Chl^R$, $Kan^R$ |
| *miu* BAC Δ*miuE* | *miu* BAC-derived, *miuE* gene was disrupted, $Chl^R$, $Kan^R$ |
| *miu* BAC Δ*miuF* | *miu* BAC-derived, *miuF* gene was disrupted, $Chl^R$, $Kan^R$ |
| *miu* BAC Δ*miuG* | *miu* BAC-derived, *miuG* gene was disrupted, $Chl^R$, $Kan^R$ |
| *miu* BAC Δ*orf25-29* | p17-9A-derived, harbouring *orf25-29* genes-disrupted *miu* cluster, 5TA, $Kan^R$ |
| *miu* BAC Δ*orf25-29&1-10* | *miu* BAC Δ*orf25-29*-derived, *orf1-10* genes were disrupted, $Chl^R$, $Kan^R$ |
| *miu* BAC Δ*orf25-29&14-16* | *miu* BAC Δ*orf25-29*-derived, *orf14-16* genes were disrupted, $Chl^R$, $Kan^R$ |
| *miu* BAC Δ*orf25-29&19-23* | *miu* BAC Δ*orf25-29*-derived, *orf19-23* genes were disrupted, $Chl^R$, $Kan^R$ |

**Table S3.** Primers used in this study.

| Primer | Sequence |
|---|---|
| pCC-BlnI 2 F | atgcctaggatcctctagagtcgacctg |
| pCC-BlnI 2 R | atgcctaggatccccgggtaccgagctc |
| miuBGC p F | caaccaagacggtcagagca |
| miuBGC p R | ctgaacccgaacgaactcac |
| loTA Gib F | aacagtgagaggttcgggtgg |
| loTA Gib R | cacggcgaacatccggaagaac |
| ploTA F | ccacccgaacctctcactgtt |
| ploTA R | tcttccggatgttcgccgtgcctttttcaattcagaagaactcgtcaag |
| ploTA check F | ttctccgccgagtagacgaa |
| ploTA check R | cattaggcaccccaggcttt |
| ploTA-Kan red F | agcagtgacaggccgtagaggcttccgatcgccgagatcggaatgagcataacagtgagaggttcgggtg |
| ploTA-Kan red R | cctgttgataccgggaagccctgggccaacttttggcgaaaatgagacgtggcgcaagggctgctaaagg |
| T1PKS1 cF | ggcgaaggcgtggaggaatt |
| T1PKS1 cR | cagttggcggcggcatagtt |
| T1PKS2 cF | gacttcttcgtgctgttctcctctt |
| T1PKS2 cR | gaagcgacaaccgataccgatgat |
| NRPS cF | gctggctgttcgttctggtcatc |
| NRPS cR | tggcggtggtgaatcggaagt |
| kanf | cgtcaagaaggcgatagaagg |
| pCC1BAC R | accgttctgtccgtcacttc |
| loTA cF | agccgaagcgataggtgag |
| loTA cR | cactgccgttgttgaggag |
| loupF | aagtgctggttcggctcggagtagttc |
| loupR | ttcagcatcgcaaccgcatcagactcc |
| 0lodownF | gcatcgagctggtggaatttctggaacagc |
| 2lodownR | agcagcaccacaccttcttcaccatcc |
| Cm-miuD-F | ccacctgcggcgaatcctccggaccaacgtgcgcaactatccaaaggcggtattcaggcgtagcaaccaggcg |
| Cm-miuD-R | ggtcacgaacggcaccacgttcgtgttctcgcccgctcgcacccggactcgttgatcggcacgtaagaggttccaa |
| Cm-miuE-F | cctgataggagacgatctcgaagccggatgtctcgaccgcattgcggtagtattcaggcgtagcaaccaggcg |
| Cm-miuE-R | gggattccaccccgtgcatcggtacagcgtcgcgttcatcgaattcctctgttgatcggcacgtaagaggttccaa |
| Cm-miuF-F | agacgaggctgcgcgagcccccactcaccgagatgagcgcgctcgtgagttattcaggcgtagcaaccaggcg |
| Cm-miuF-R | catggtgccgaaccacgcggcgatcggtacttcgagcggtgcctcggcaagttgatcggcacgtaagaggttccaa |
| Cm-miuG-F | gttcgaggctctgctcggcgagttcgggcgcggagtctggaccggtgaggtattcaggcgtagcaaccaggcg |
| Cm-miuG-R | atccgcagccggatccacctagagcatcgggtcgagcaggcgcacttcgagttgatcggcacgtaagaggttccaa |
| 5TA-orf25-F | agtagatcttcacgcccgcacccaggtgcatcgcggcatcgacgtcgttgaacagtgagaggttcgggtggt |
| Cm-orf1-F | tcgaggacgaggtactgcatgcgctcttcgaacagttcgtggtcgagcattattcaggcgtagcaaccaggcg |
| Cm-orf10-R | acgaccgaccacacgtttggccacacgtttggcccgcccactggccaaatgttgatcggcacgtaagaggttccaa |
| Cm-orf14-F | gacgaatgcctcgagcagcaggcggttgagcgcattcttgatctcgaagttattcaggcgtagcaaccaggcg |
| Cm-orf16-R | gccgagatcgaccagcagttcgatctgtcgctcgtgtgcctgggcatcaagttgatcggcacgtaagaggttccaa |
| Cm-orf19-F | atcactcttgtcgtcgctcaatcggtccaagaggtcaccggtgaagggcctattcaggcgtagcaaccaggcg |
| Cm-orf23-R | tgagctggtggaccgagctgcgggattttggactcgagctcatccaggatgttgatcggcacgtaagaggttccaa |
| Cm cF | ggtattcactccagagcgat |

| | |
|---|---|
| Cm cR | agacggtgagctggtgatatgg |
| dmiuD cF | cgtgaagatgccgctgtggaa |
| dmiuD cR | cctgccacgattctcatcg |
| dmiuE cR | accagacgacaccgacaa |
| miuEF | gctcttcaagtacgcctcca |
| dmiuF cF | cagacacaacgaacgacgaac |
| dmiuF cR | agcaagccgagcgagatt |
| dmiuG cF | atggtgacgagtggcggtag |
| miuGR | cgctcaaggagttcactgc |
| orf24 cF | cgaactatgccgatgaggaga |
| dorf1 cF | agtggtgaggtgcttccga |
| dorf10 cR | ggctggcaacctacactt |
| dorf14 cF | cctcgaactcgatgcccttc |
| dorf16 cR | cgcagggaatccaaggtt |
| dorf19 cF | tcggcttgtagtcaacggatg |
| dorf23 cR | accatgacggcgatgaac |

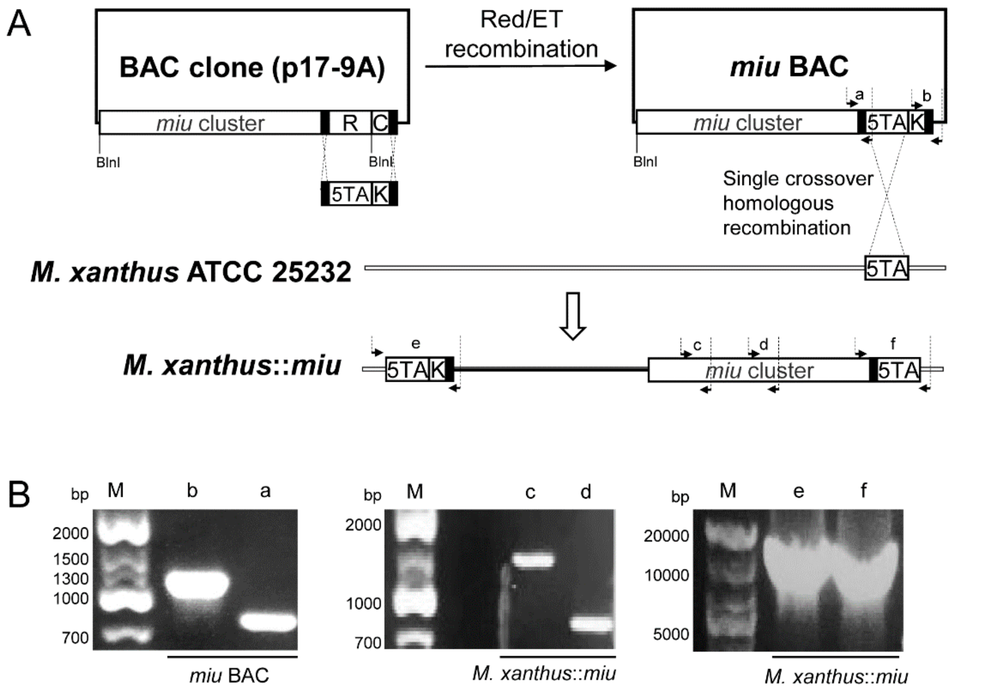


**Figure S1.** Construction of *M. xanthus*::*miu*. (A) The redundant fragment (genomic fragment outside the *miu* cluster) and chloramphenicol resistance gene (R|C) in the BAC clone p17-9A was replaced with the cassette 5TA-Kan$^R$ (5TA|K, amplified from p5TA-Kan$^R$ with primer pair ploTA-Kan red F/R) by Red/ET recombination. The generated *miu* BAC was integrated into the genome of *M. xanthus* ATCC 25232 by homologous recombination via the 5TA region. (B) PCR verification of the constructed vector and mutant by the following primer pairs: a (loTA cF/loTA cR, 886 bp), b (kanf/pCC1BAC R, 1299 bp), c (T1PKS1 cF/T1PKS1 cR, 1274 bp), d (T1PKS2 cF/T1PKS2 cR, 783 bp), e (loupF/loupR, 7957 bp), and f (0lodownF/2lodownR, 7488). M: DNA marker (Gene Ladder Wide 1, Nippon Gene).

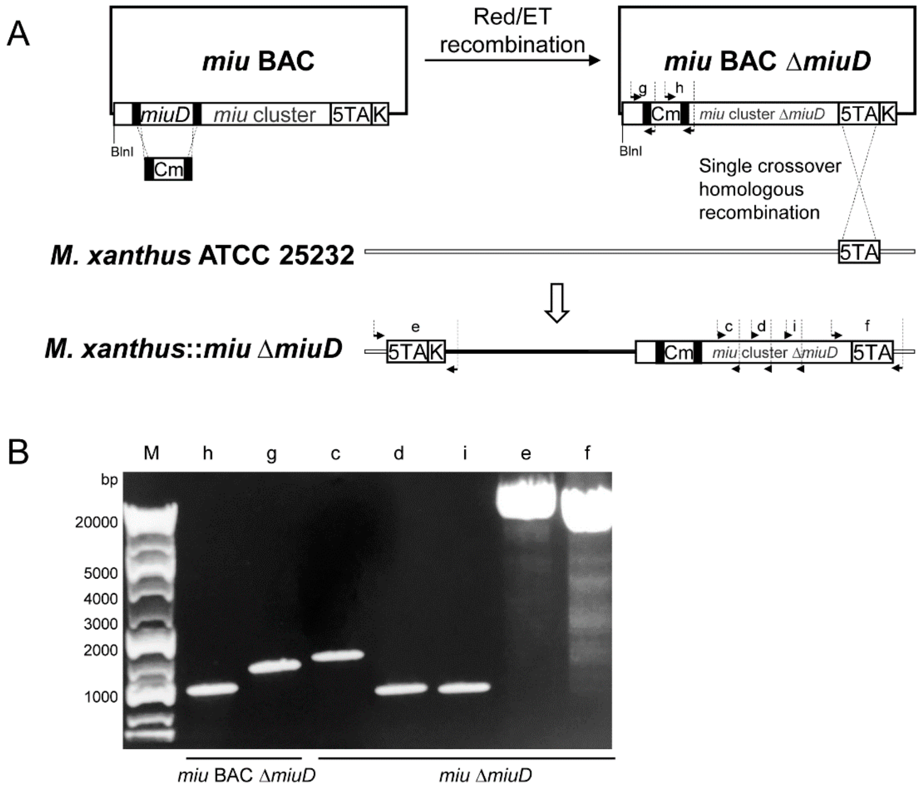


**Figure S2.** Construction of *M. xanthus*::*miu* Δ*miuD*. (A) The gene *miuD* in *miu* BAC was replaced with the chloramphenicol resistance gene (Cm, amplified from pCCIBAC with primer pair Cm-miuD-F/R) by Red/ET recombination. The generated *miu* BAC Δ*miuD* was integrated into the genome of *M. xanthus* ATCC 25232 by homologous recombination via the 5TA region. (B) PCR verification of the constructed mutants by the following primer pairs: g (dmiuD cF/Cm cR, 1186 bp), h (Cm cF/dmiuD cR, 949 bp), c (T1PKS1 cF/T1PKS1 cR, 1274 bp), d (T1PKS2 cF/T1PKS2 cR, 783 bp), I (NRPS cF/NRPS cR, 756 bp), e (loupF/loupR, 7957 bp), and f (0lodownF/2lodownR, 7488 bp). M: DNA marker.

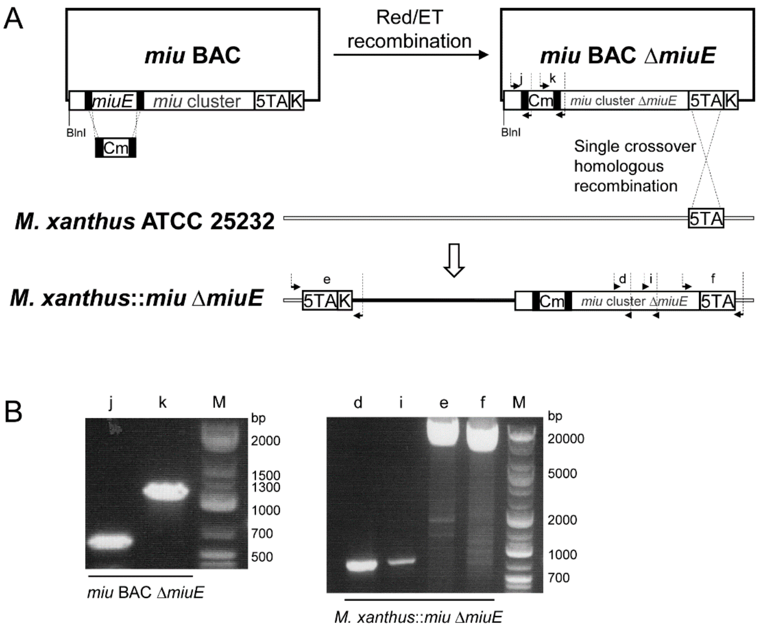


**Figure S3.** Construction of *M. xanthus*::*miu* Δ*miuE*. (A) The gene *miuE* in *miu* BAC was replaced with the chloramphenicol resistance gene (Cm, amplified from pCCIBAC with primer pair Cm-miuE-F/R) by Red/ET recombination. The generated *miu* BAC Δ*miuE* was integrated into the genome of *M. xanthus* ATCC 25232 by homologous recombination via the 5TA region. (B) PCR verification of the constructed mutants by the following primer pairs: j (miuEF/Cm cR, 599 bp), k (Cm cF/dmiuE cR, 1037 bp), d (T1PKS2 cF/T1PKS2 cR, 783 bp), i (NRPS cF/NRPS cR, 756 bp), e (loupF/loupR, 7957 bp), and f(0lodownF/2lodownR, 7488 bp). M: DNA marker.

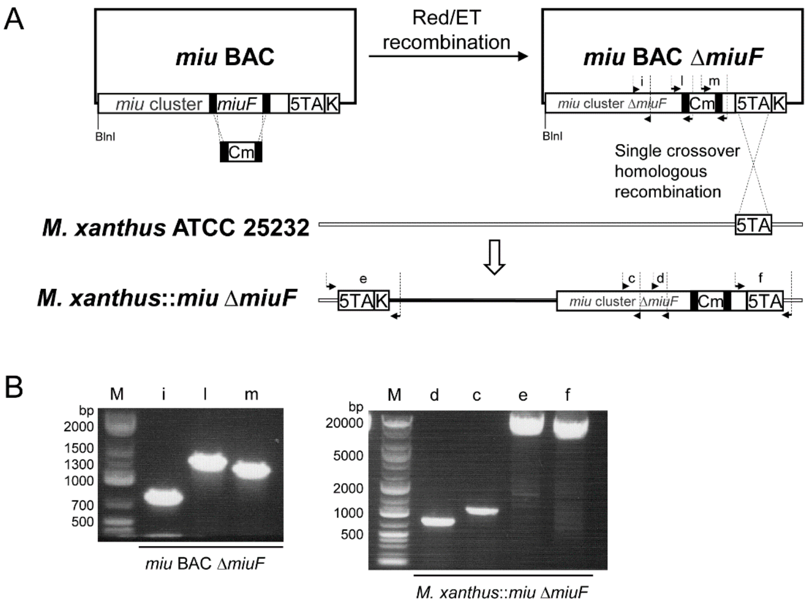


**Figure S4.** Construction of *M. xanthus*::*miu* Δ*miuF*. (A) The gene *miuF* in *miu* BAC was replaced with the chloramphenicol resistance gene (Cm, amplified from pCCIBAC with primer pair Cm-miuF-F/R) by Red/ET recombination. The generated *miu* BAC Δ*miuF* was integrated into the genome of *M. xanthus* ATCC 25232 by homologous recombination via the 5TA region. (B) PCR verification of the constructed mutants by the following primer pairs: i (NRPS cF/NRPS cR, 756 bp), l (dmiuF cF/Cm cR, 1201 bp), m (Cm cF/dmiuF cR, 1121 bp), c (T1PKS1 cF/T1PKS1 cR, 1274 bp), d (T1PKS2 cF/T1PKS2 cR, 783 bp), e (loupF/loupR, 7957 bp), and f (0lodownF/2lodownR, 7488 bp). M: DNA marker.

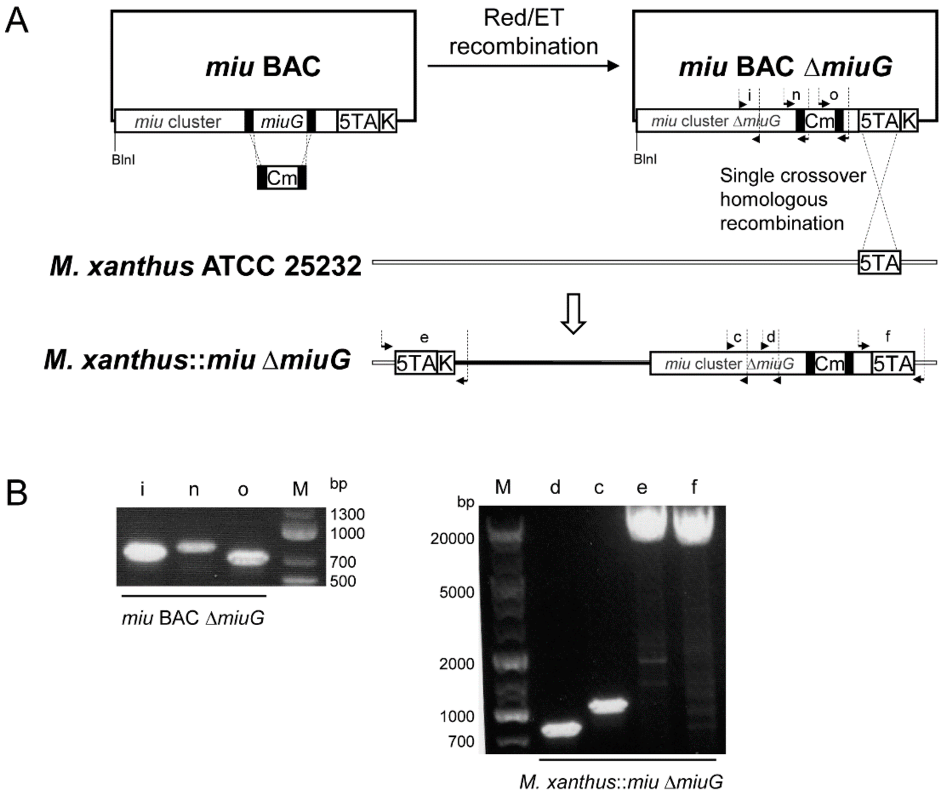


**Figure S5.** Construction of *M. xanthus*::*miu* Δ*miuG*. (A) The gene *miuG* in *miu* BAC was replaced with the chloramphenicol resistance gene (Cm, amplified from pCCIBAC with primer pair Cm-miuG-F/R) by Red/ET recombination. The generated *miu* BAC Δ*miuG* was integrated into the genome of *M. xanthus* ATCC 25232 by homologous recombination via the 5TA region. (B) PCR verification of the constructed mutants by the following primer pairs: i (NRPS cF/NRPS cR, 756 bp), n (dmiuG cF/Cm cR, 778 bp), o (Cm cF/miuGR, 701 bp), c (T1PKS1 cF/T1PKS1 cR, 1274 bp), d (T1PKS2 cF/T1PKS2 cR, 783 bp), e (loupF/loupR, 7957 bp), and f (0lodownF/2lodownR, 7488 bp). M: DNA marker.

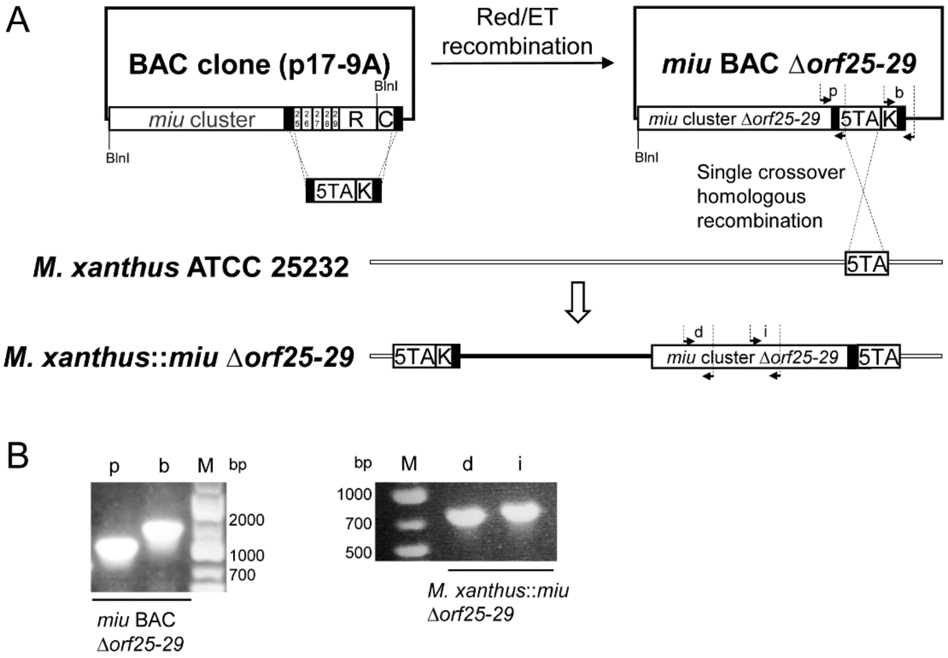


**Figure S6.** Construction of *M. xanthus*::*miu* Δ*orf25-29*. (A) The gene region *orf25-29*, the redundant fragment and chloramphenicol resistance gene (*25*|*26*|*27*|*28*|*29*|R|C), in the BAC clone p17-9A was replaced with the cassette 5TA-Kan$^R$ (5TA|K, amplified from p5TA-Kan$^R$ with primer pair 5TA-orf25-F/ploTA-Kan red R) by Red/ET recombination. The generated *miu* BAC Δ*orf25-29* was integrated into the genome of *M. xanthus* ATCC 25232 by homologous recombination via the 5TA region. (B) PCR verification of the constructed mutants by the following primer pairs: p (orf24 cF/loTA cR, 1020 bp), b (kanf/pCC1BAC R, 1299 bp), d (T1PKS2 cF/T1PKS2 cR, 783 bp), and i (NPRS cF/NRPS cR, 756 bp). M: DNA marker.

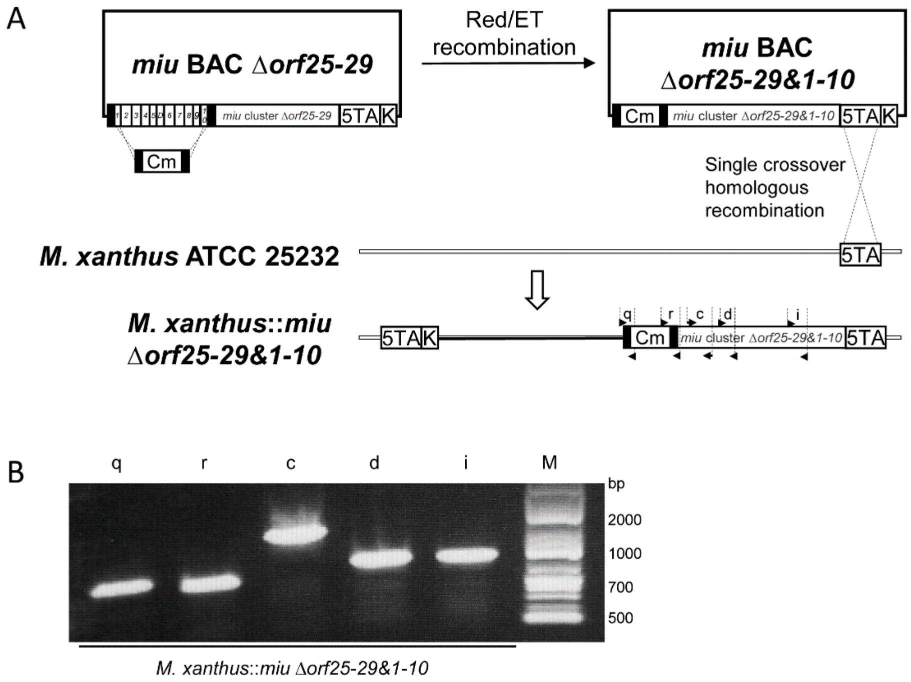


**Figure S7.** Construction of *M. xanthus*::*miu* Δ*orf25-29&1-10*. (A) The gene region *orf1-10* (*1*|*2*|*3*|*4*|*5*|*D*|*6*|*7*|*8*|*9*|*10*) in *miu* BAC Δ*orf25-29* was replaced with the chloramphenicol resistance gene (Cm, amplified from pCCIBAC with primer pair Cm-orf1-F/ Cm-orf10-R) by Red/ET recombination. The generated *miu* BAC Δ*orf25-29&1-10* was integrated into the genome of *M. xanthus* ATCC 25232 by homologous recombination via the 5TA region. (B) PCR verification of *M. xanthus*::*miu* Δ *orf25-29&1-10* by the following primer pairs: q (dorf1 cF/Cm cR, 694 bp), r (Cm cF/dorf10 cR, 621 bp), c (T1PKS1 cF/T1PKS2 cR, 1274 bp), d (T1PKS2 cF/T1PKS2 cR, 783 bp), and i (NPRS cF/NRPS cR, 756 bp). M: DNA marker.

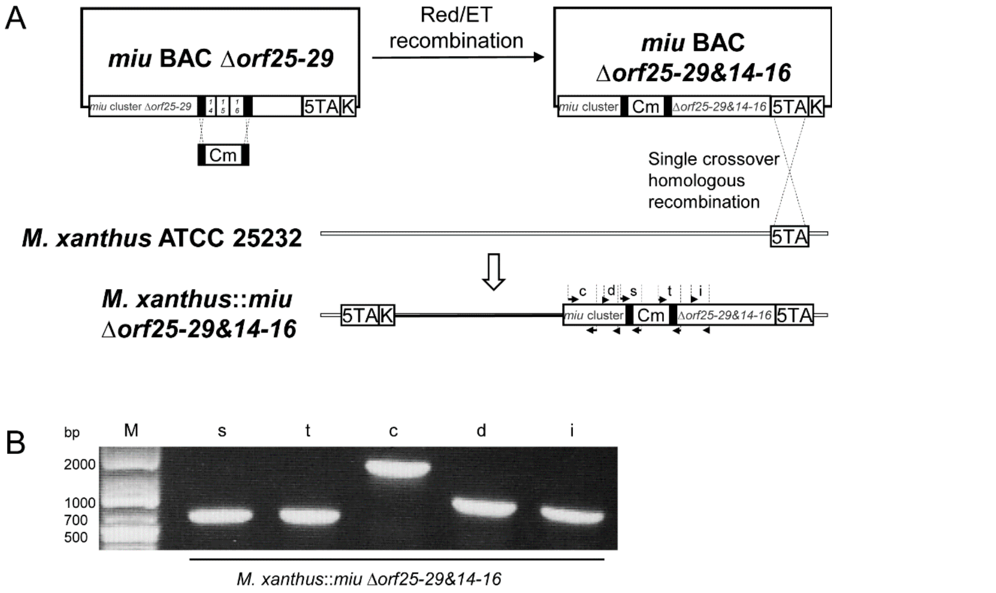


**Figure S8.** Construction of *M. xanthus*::*miu* Δ*orf25-29&14-16*. (A) The gene region *orf14-16* (*14*|*15*|*16*) in *miu* BAC Δ*orf25-29* was replaced with the chloramphenicol resistance gene (Cm, amplified from pCCI-BAC with primer pair Cm-orf14-F/ Cm-orf16-R) by Red/ET recombination. The generated *miu* BAC Δ*orf25-29&14-16* was integrated into the genome of *M. xanthus* ATCC 25232 by homologous recombination via the 5TA region. (B) PCR verification of *M. xanthus*::*miu* Δ *orf25-29&14-16* by the following primer pairs: s (dorf14 cF/Cm cR, 577 bp), t (Cm cF/dorf16 cR, 591 bp), c (T1PKS1 cF/T1PKS2 cR, 1274 bp), d (T1PKS2 cF/T1PKS2 cR, 783 bp), and i (NPRS cF/NRPS cR, 756 bp). M: DNA marker.

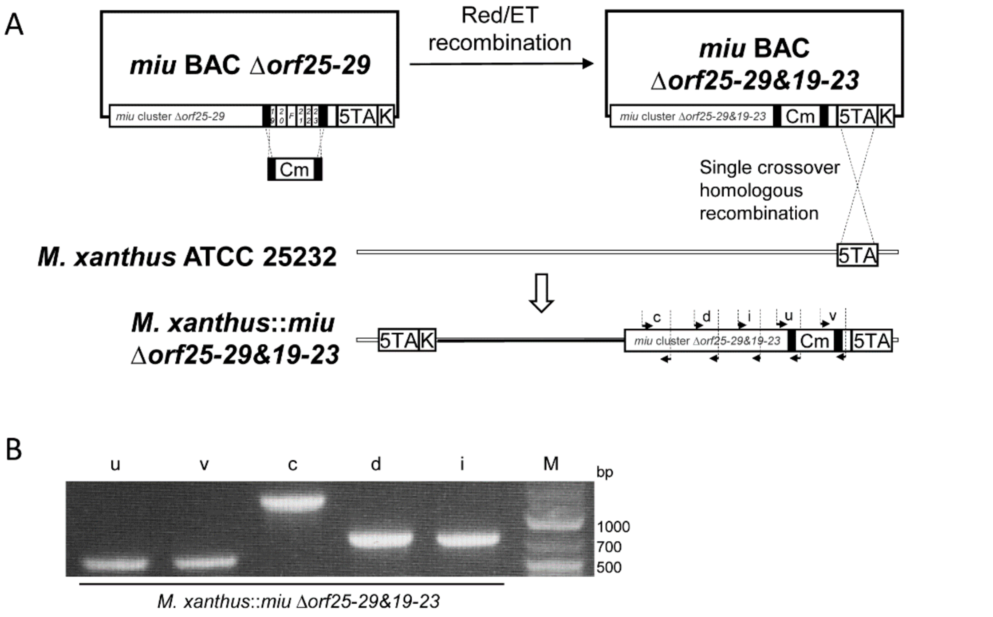


**Figure S9.** Construction of *M. xanthus*::*miu* Δ*orf25-29&19-23*. (A) The gene region *orf19-23* (*19*|*20*|*F*|*21*|*22*|*23*) in *miu* BAC Δ*orf25-29* was replaced with the chloramphenicol resistance gene (Cm, amplified from pCCIBAC with primer pair Cm-orf19-F/ Cm-orf23-R) by Red/ET recombination. The generated *miu* BAC Δ*orf25-29&19-23* was integrated into the genome of *M. xanthus* ATCC 25232 by homologous recombination via the 5TA region. (B) PCR verification of *M. xanthus*::*miu* Δ *orf25-29&19-23* by the following primer pairs: u (dorf19 cF/Cm cR, 582 bp), v (Cm cF/dorf23 cR, 576 bp), c (T1PKS1 cF/T1PKS2 cR, 1274 bp), d (T1PKS2 cF/T1PKS2 cR, 783 bp), and i (NPRS cF/NRPS cR, 756 bp). M: DNA marker.

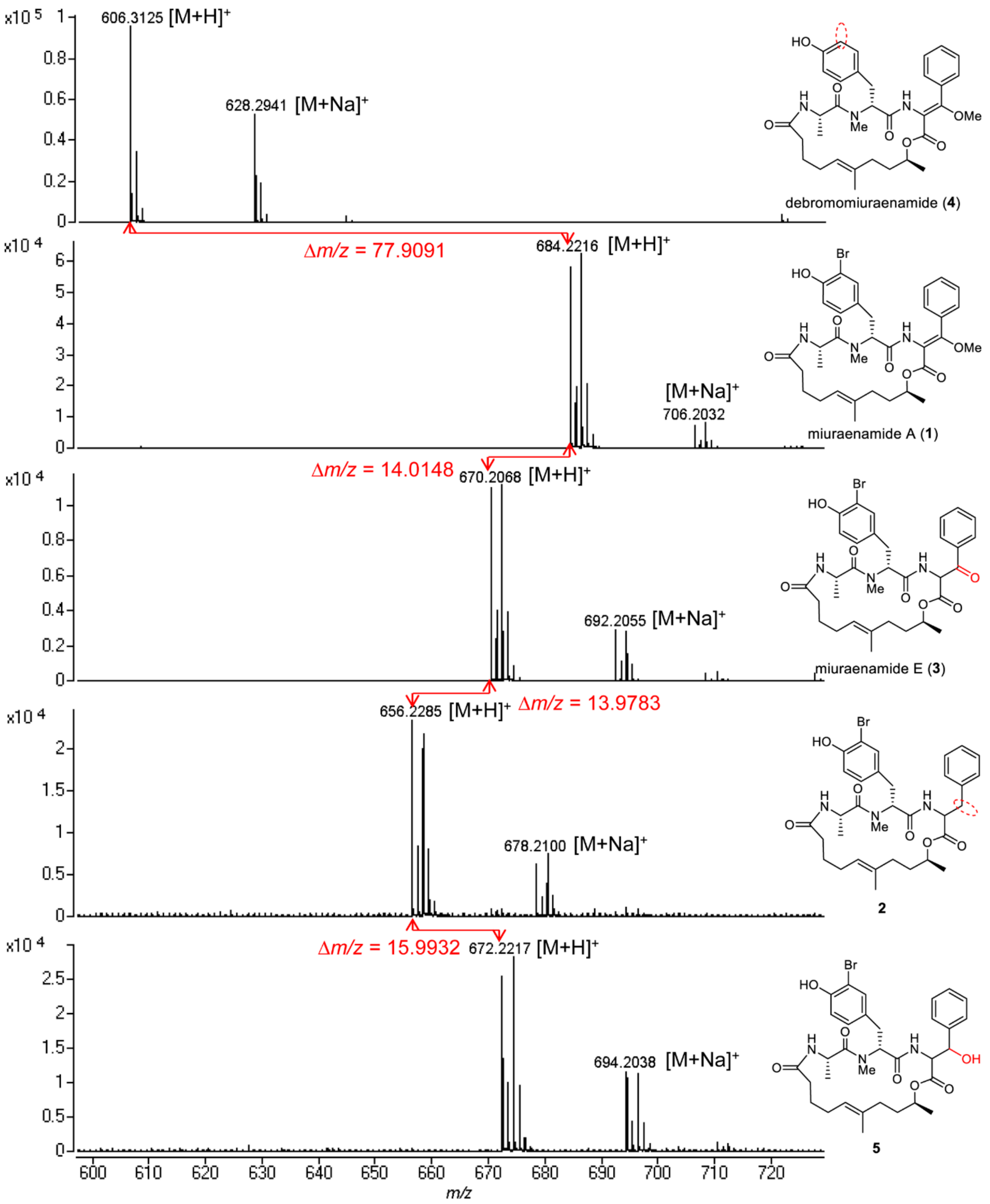


**Figure S10.** Mass spectra of miuraenamide A and its congeners. The structures of **2**, **4**, **5** are estimated by the molecular formulae compared with those of **1** and **3** and not verified by NMR.

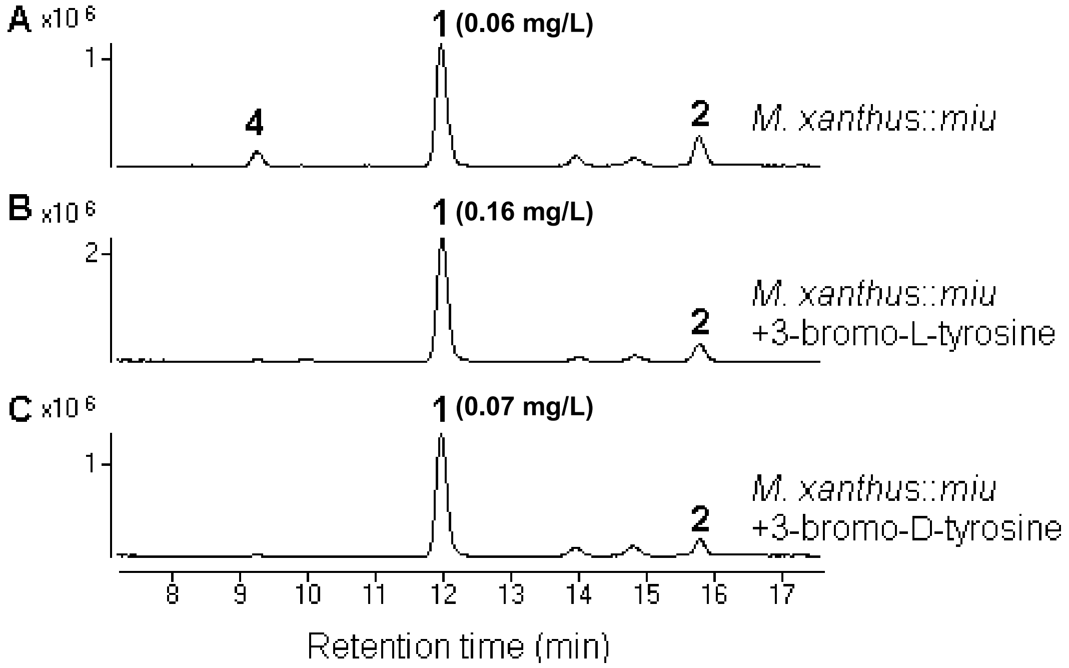


**Figure S11.** Production of miuraenamide A (**1**) and its congeners by *M. xanthus*::*miu* mutant fed on bromotyrosines. Extracted ion chromatographs (merged from *m*/*z* 684.2216, 606.3125, 656.2285, 670.2068, and 672.2217) of the extracts of the heterologous producer *M. xanthus*::*miu* (A), fed on 3-bromo-L-tyrosine (B), fed on 3-bromo-D-tyrosine (C).

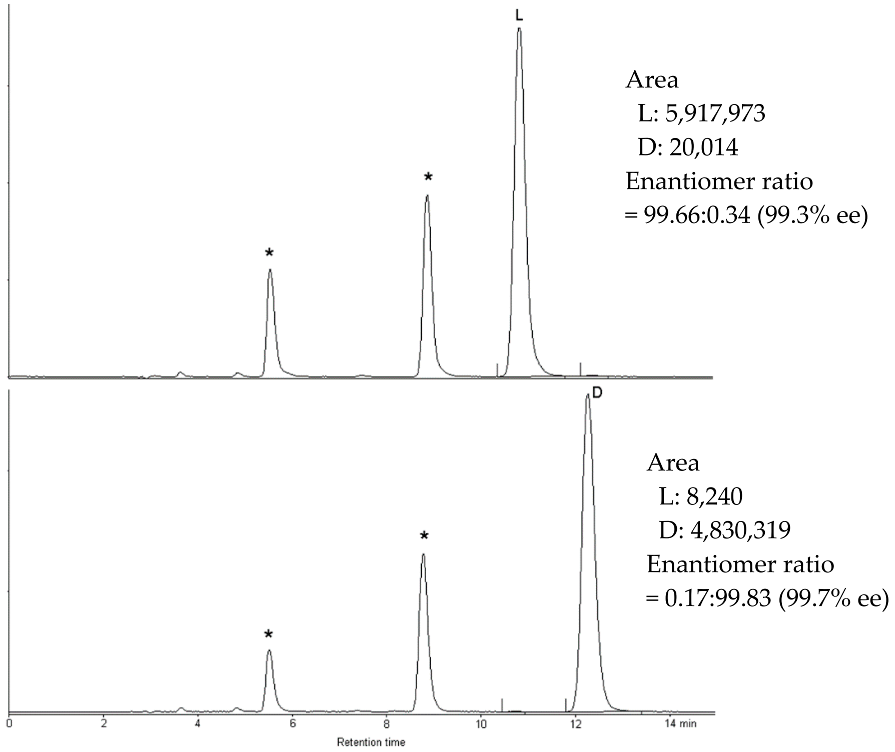


**Figure S12.** Enantiomeric purity of 3-bromo-D and L-tyrosines determined by Marfey's method. Column: Develosil ODS-UG-5 (4.6 x 250 mm), Solvent: 40% MeCN-0.1% TFA, flow: 1 mL/min, detection: 320 nm. * Impurities originated from Marfey's reagent (FDAA).
